# Benign Fibrous Histiocytoma of the Calcaneus: A Case Report of a Rare Bone Tumor

**DOI:** 10.7759/cureus.80635

**Published:** 2025-03-15

**Authors:** Abheek Kar, Soumyadip Sen, Abhishek Das, Arpita Sutradhar

**Affiliations:** 1 Orthopedics, Apollo Multispeciality Hospitals, Kolkata, IND; 2 Pathology, Apollo Multispeciality Hospitals, Kolkata, IND

**Keywords:** benign fibrous histiocytoma, bone tumors, calcaneal tumor, calcaneus, fibular strut graft

## Abstract

A benign fibrous histiocytoma (BFH) is a rare tumor that typically occurs in the pelvis and non-metaphyseal regions of long bones, making its presence in the calcaneus extremely unusual. We present the case of a 42-year-old male who experienced right heel pain for a year, initially worsening with weight-bearing on the right leg. Over the past month, the pain began occurring even at rest and was only relieved by over-the-counter analgesics. Clinical examination revealed a heterogeneous, tender swelling of the right heel without erythema or venous prominence. The calcaneal squeeze test triggered moderate to severe pain. Standard radiographs showed a cystic lesion occupying nearly the entire calcaneus, accompanied by cortical thinning and breaching. MRI displayed a lobulated area with altered signal intensity, appearing mildly hypointense on T1-weighted sequences and hyperintense on T2-weighted sequences, measuring 51 mm × 31 mm × 39 mm. A core needle biopsy from the lesion identified a few multinucleated giant cells without signs of mitosis or necrosis. After thorough discussion and obtaining informed consent, we performed an extended curettage of the lesion. We then placed ipsilateral autologous non-vascularized fibular strut grafts into the cavity, supplemented by autologous tricortical iliac crest grafts and synthetic hydroxyapatite granules as artificial bone grafts. Histopathological examination of the excised tissue revealed fascicles of spindle cells with elongated vesicular nuclei and moderate cytoplasm, interspersed with multinucleated giant cells. Correlating the clinical, radiological, and histopathological findings confirmed the diagnosis of BFH of the calcaneus. The patient achieved full weight-bearing mobility three months post-surgery. At the five-year follow-up, he remained pain-free with no signs of local recurrence and demonstrated a full range of motion in the right ankle. His American Orthopaedic Foot & Ankle Society (AOFAS) ankle-hindfoot score improved significantly from 55 at the initial visit to 100 at the final follow-up. BFH rarely affects the calcaneus, with this being only the third reported case. Due to the extensive involvement of the calcaneus, using autologous fibular strut grafts provided enhanced stability and mechanical strength. This approach proved to be more cost-effective than allogeneic bone grafts and avoided the need for additional stabilization with implants.

## Introduction

A benign fibrous histiocytoma (BFH) is an uncommon skeletal tumor, representing approximately 1% of all benign bone tumors that undergo surgical treatment [1]. While BFH can develop in any bone, it most commonly occurs in the non-metaphyseal regions of long bones and the pelvis [2,3]. Its presence in the calcaneus is exceptionally rare.

Radiographically, BFH can resemble other conditions such as simple bone cysts, intraosseous lipomas, or giant cell tumors [3]. Additionally, its histopathological features often overlap with those of non-ossifying fibromas and fibrous cortical defects [2,3]. Therefore, clinical correlation is essential, and careful consideration is required when diagnosing BFH in atypical locations. The preferred treatment approach is complete surgical excision followed by reconstruction of the resulting bone defect [3,4].

In this report, we present a case of primary BFH in the calcaneus, managed through extended curettage and reconstruction using autologous non-vascularized fibular strut grafts, tricortical iliac crest graft, and synthetic hydroxyapatite granules as artificial bone grafts.

## Case presentation

A 42-year-old averagely built male without known comorbidities presented with pain in the right heel lasting for one year and swelling for six months. There was no history of specific trauma associated with the onset of symptoms. Initially, the pain occurred only during running and was relieved with rest. However, the pain intensity gradually increased over the following five months. The heel swelling developed insidiously and worsened as the pain intensified.

Over the past month, the pain occurred even at rest and was relieved only by over-the-counter pain-relieving medications, which he required on most days of the week. There was no history of significant weight loss, reduced appetite, or a family history of skeletal tumors.

A regional examination revealed a heterogeneous, tender swelling over the right heel. The overlying skin appeared normal, with no erythema or venous prominence. Tenderness was noted over the lateral and plantar surfaces of the right heel, and the calcaneal squeeze test elicited moderate to severe pain. The ankle’s range of motion was full and painless, with no signs of neurovascular compromise. The American Orthopaedic Foot & Ankle Society (AOFAS) ankle-hindfoot score was 55 out of a maximum score of 100.

**Figure 1:**
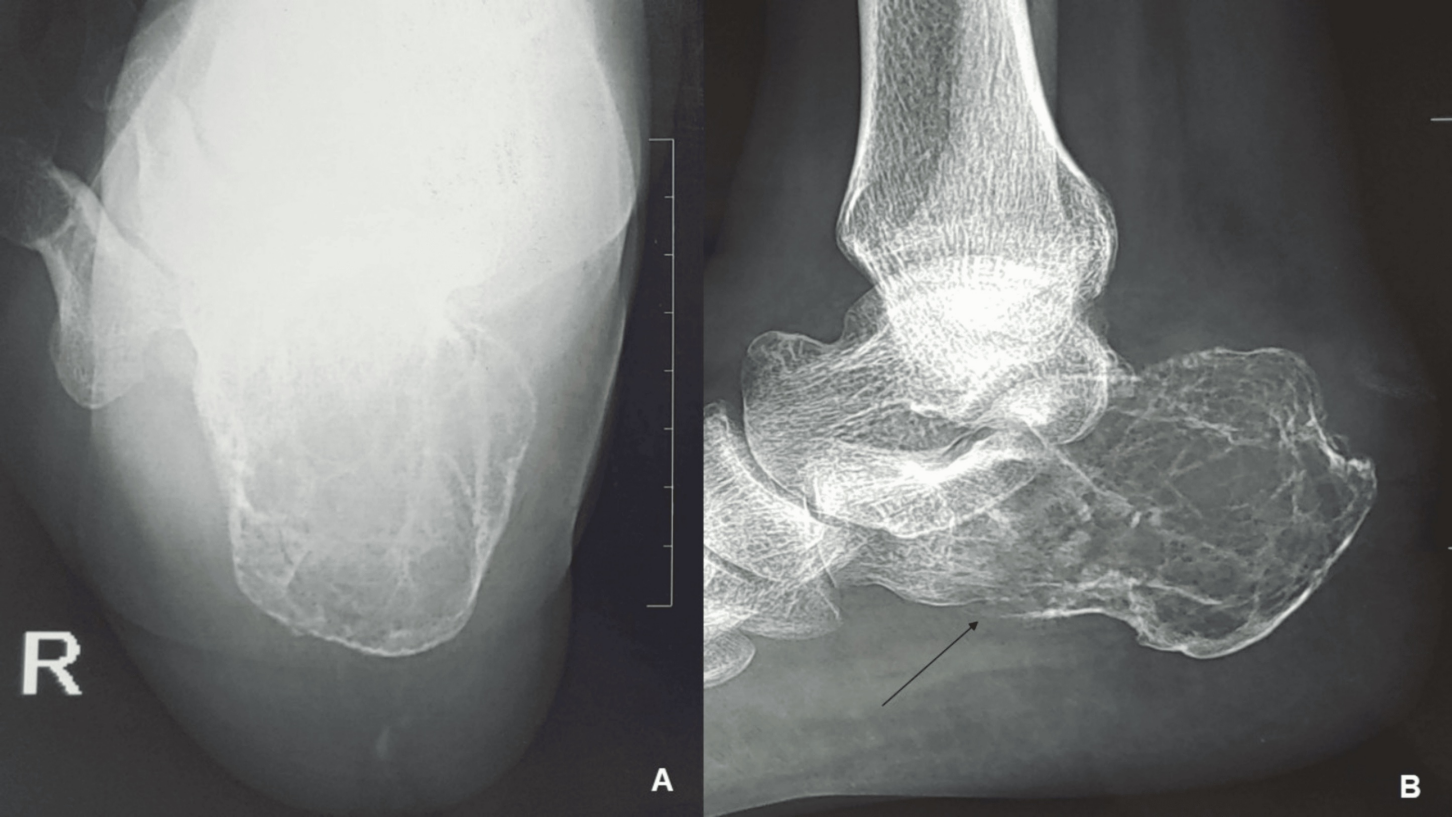
Plain radiographs of the right calcaneus (A) Axial view of the calcaneus showing a cystic lesion. (B) Lateral radiograph illustrating the cystic lesion involving nearly the entire right calcaneus, with a black arrow indicating the cortical breach.

**Figure 2:**
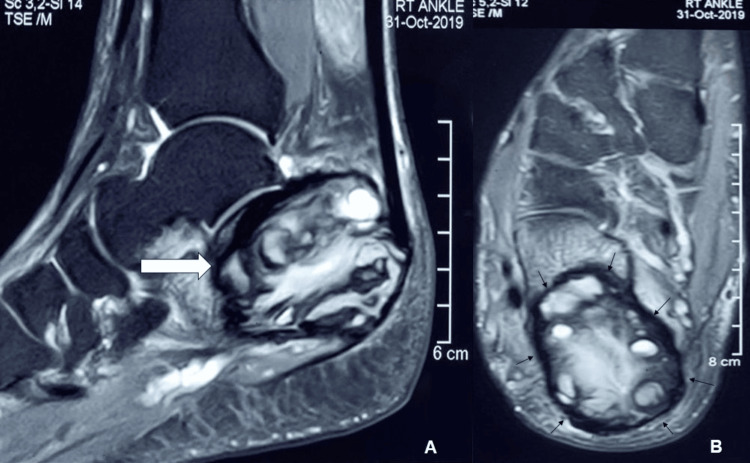
MRI of the right calcaneus (A) Sagittal image showing the mass with a lobulated margin, indicated by an arrow. (B) Axial image illustrating the extent of the lesion involving nearly the entire calcaneus, with arrows highlighting the affected areas.

Laboratory investigations revealed no significant abnormalities. Standard radiographs showed a cystic lesion involving nearly the entire calcaneus, with thinning of the cortex and a cortical breach on the undersurface, but no cortical expansion (Figure 1A, 1B). MRI demonstrated a lobulated margin area with altered signal intensity, appearing mildly hypointense on T1-weighted images and hyperintense on T2-weighted images. The lesion measured 51 mm in the anteroposterior plane, 31 mm in the mediolateral plane, and 39 mm in the supero-inferior plane (Figure 2A, 2B). It was associated with thinning of the calcaneal cortex, with cortical breach in some areas. A PET-CT scan revealed no evidence of a primary tumor elsewhere or metastasis.

The patient was initially placed in a temporary back slab to prevent further progression of a pathological fracture and was allowed to mobilize with crutches. A core needle biopsy of the lesion was performed, revealing a few multinucleated giant cells without evidence of mitosis or necrosis. Treatment options were discussed with the patient, and written informed consent was obtained for lesion excision followed by bone grafting.

Given the anticipated large post-curettage calcaneal defect and the high cost and unavailability of allografts due to the lack of a bone bank, we opted to use autologous non-vascularized fibular strut grafts. This approach also helped avoid potential complications associated with the use of bone cement.

**Figure 3:**
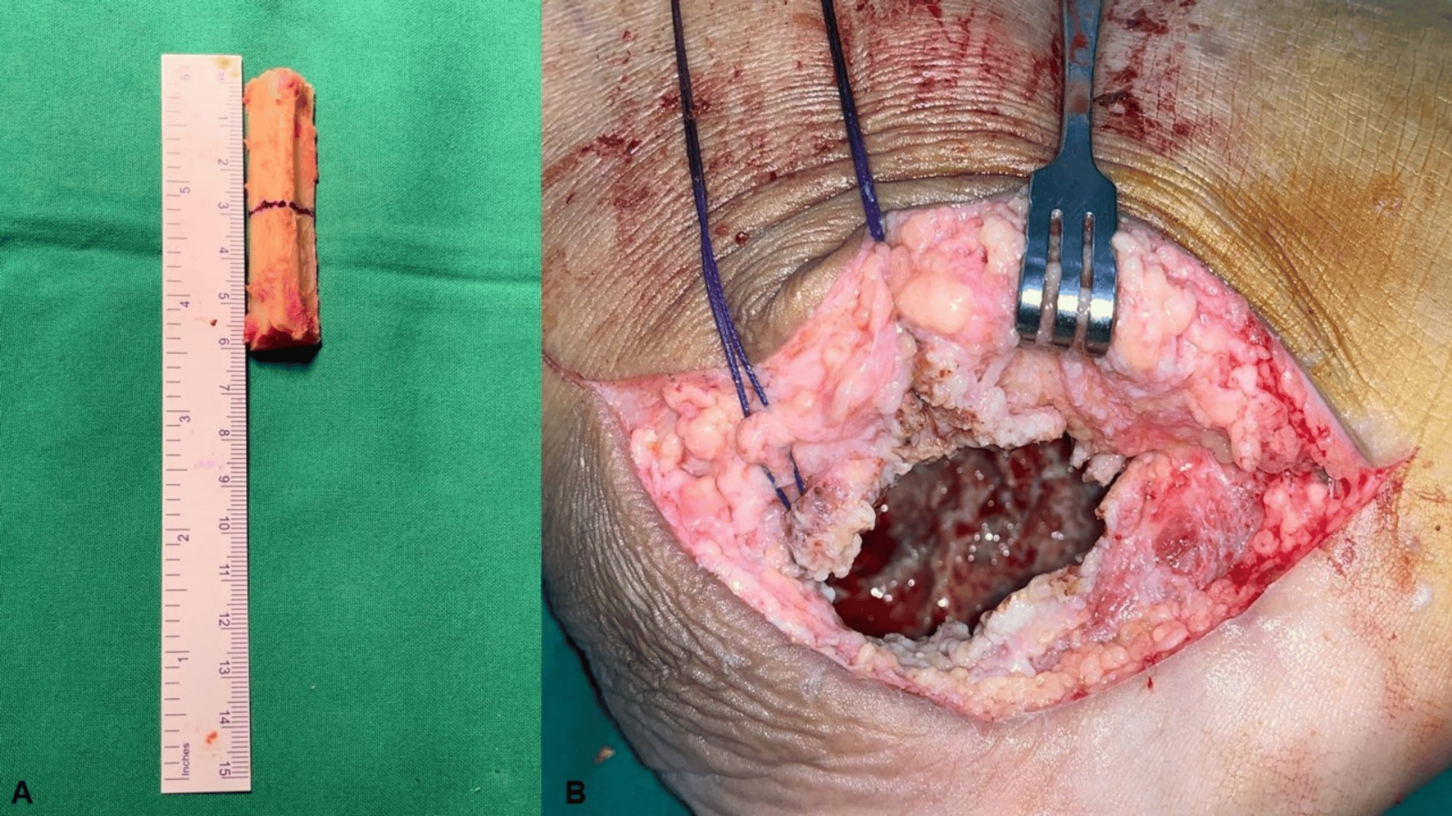
Intraoperative images (A) Free fibular strut autografts harvested from the ipsilateral fibula. (B) Calcaneal cavity following complete removal of tumor tissue.

Under spinal anesthesia, an autologous tricortical bone graft was harvested from the ipsilateral iliac crest, while fibular strut grafts were obtained from the ipsilateral fibula (Figure 3A). The calcaneus was accessed via a lateral approach, and a cortical window was created to expose the tumor. The tumor tissue was meticulously scraped out until healthy, bleeding bone was observed (Figure 3B).

A phenol solution was applied to eliminate residual tumor cells, followed by thorough drainage and rinsing with water. The fibular strut grafts were then inserted into the calcaneal cavity, and the remaining cavity was packed with tricortical iliac crest graft pieces and artificial bone graft in the form of synthetic hydroxyapatite granules. Finally, the cortical window was repositioned like a trap door.

**Figure 4:**
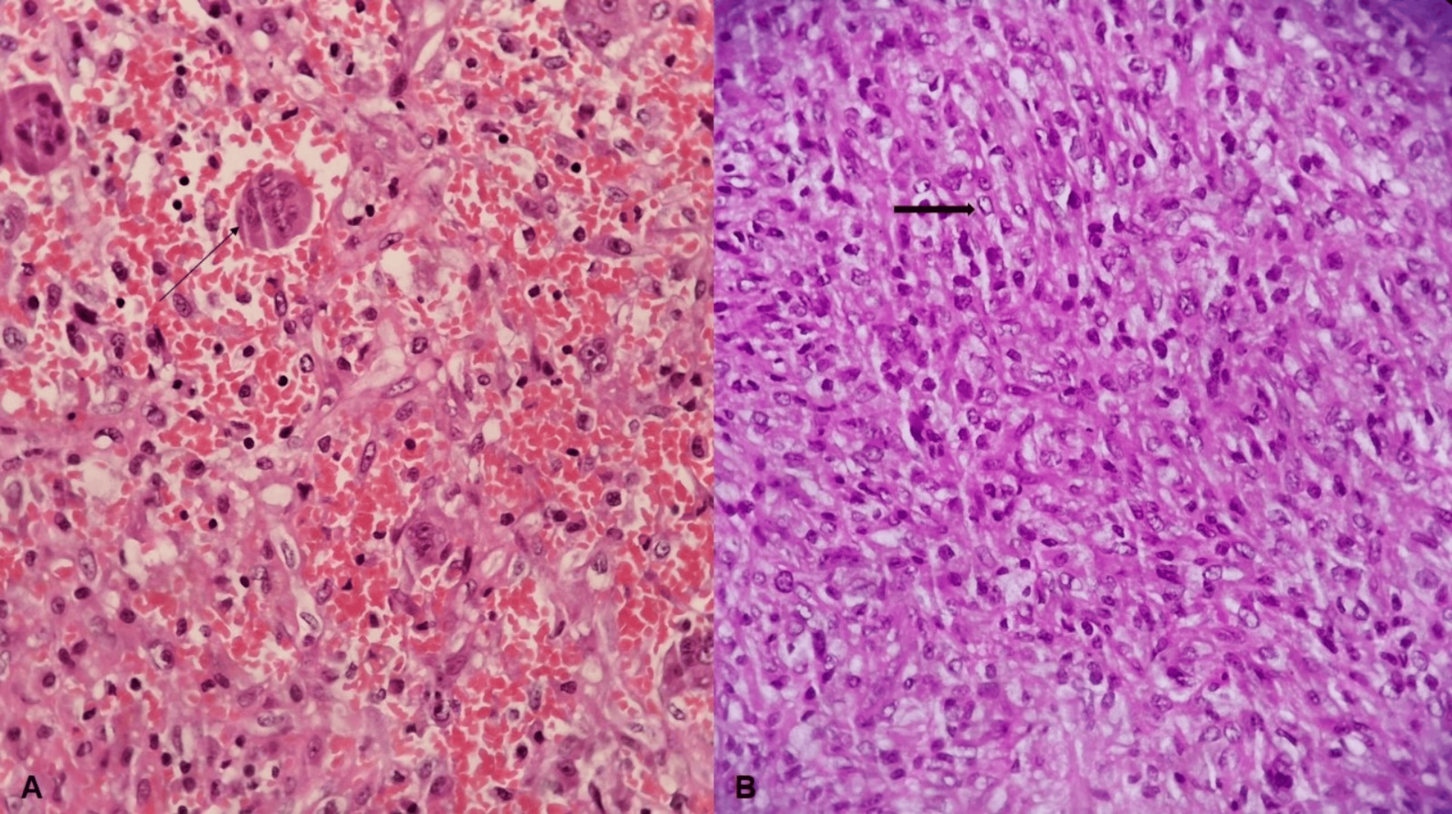
Histopathological examination of the tumor tissue (A) H&E-stained slide at 40× magnification, with an arrow indicating a multinucleated giant cell. (B) H&E-stained slide at 40× magnification, with an arrow indicating a spindle cell; note the absence of mitosis and necrosis.

Histopathological examination demonstrated fascicles of spindle cells with elongated vesicular nuclei and moderate cytoplasm, interspersed with multinucleated giant cells (Figure 4A, 4B). No evidence of mitosis or necrosis was observed. The diagnosis was confirmed as BFH of the calcaneus.

**Figure 5:**
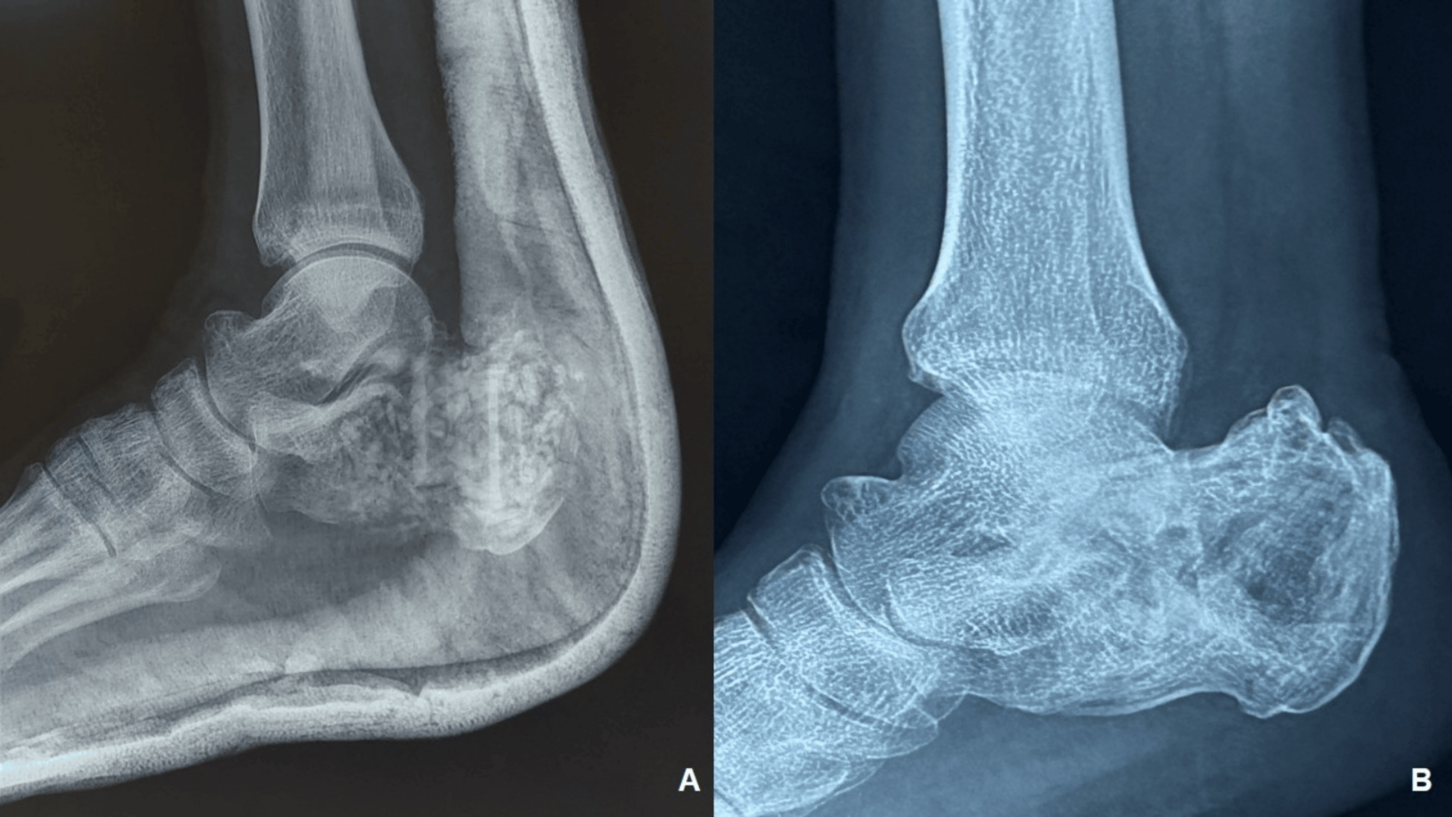
Postoperative radiographs of the right calcaneus (A) Immediate postoperative radiograph showing the absence of residual cystic lesions. (B) Radiograph at the five-year follow-up demonstrating complete graft healing with no signs of recurrence.

The leg was placed in a below-knee plaster slab, and intermittent ankle mobilization, as tolerated by the patient, was initiated from the first postoperative day. The immediate postoperative radiograph showed no evidence of residual lytic lesions (Figure 5A). Right lower limb toe-touch weight-bearing mobilization with crutch support began on the second postoperative day. Stitches were removed two weeks post-surgery, and partial weight-bearing was permitted six weeks after the procedure. Full weight-bearing was allowed after three months.

At the five-year follow-up, the patient was doing well without any symptoms, achieving an AOFAS ankle-hindfoot score of 100. He demonstrated a full range of motion at the ankle, with adequate graft healing and no signs of local recurrence (Figure 5B).

## Discussion

BFH is a tumor that typically occurs in the dermis and soft tissue, but its occurrence in bone is extremely rare [4]. It is a spindle cell neoplasm composed of varying amounts of spindle cells, multinucleated giant cells, and foam cells [3]. According to the World Health Organization’s classification of tumors, BFH is categorized under the same disease entity as non-ossifying fibroma, as both share similar microscopic features [2]. However, they can be distinguished based on clinical presentation, age at onset, and the lesion’s site of occurrence.

Patients with BFH typically present with pain, which usually occurs in adults after the second decade of life and primarily involves the pelvis and non-metaphyseal regions of long bones [2,5]. Cases have also been reported in the ribs and vertebrae [4].

Bone tumors in the foot are relatively uncommon, accounting for only 3% of all skeletal tumors. While primary malignancies originating from the calcaneus are rare, it is the second most common site for bone tumors in the foot, following the metatarsals [6,7]. Although the foot is not a typical site for metastatic lesions, when affected, the calcaneus and talus are the most commonly involved bones [8]. Common benign bone tumors occurring in the calcaneus include simple bone cysts, aneurysmal bone cysts, and osteoid osteoma [7]. BFH of the calcaneus is extremely rare, and this is only the third reported case.

Radiographically, BFH of bone usually appears as an osteolytic lesion with trabeculations and bone sclerosis [4]. In some instances, cortical expansion with or without disruption may be present. However, these radiographic findings are not exclusive to BFH and can be seen in other benign bone tumors [3]. Simple bone cysts, intraosseous lipomas, and calcaneal pseudocysts are among the most common calcaneal tumors with similar radiographic appearances, but they are often asymptomatic and typically discovered incidentally or after a pathological fracture [9]. These lesions generally affect the anterior calcaneal lacuna, also known as the neutral triangle [3].

Giant cell tumors can present with similar features but are characterized by lytic, expansile, or eccentric lesions with aggressive traits such as cortical expansion and soft tissue destruction [10]. In our patient, the lesion involved nearly the entire calcaneus with cortical disruption but lacked aggressive features.

Microscopically, BFH is characterized by spindle-shaped fibroblasts arranged in a whorled or storiform pattern, along with varying amounts of foam cells and multinucleated osteoclastic giant cells. Notably, there is an absence of atypia, mitoses, or pleomorphism [4]. This histological pattern closely resembles that of non-ossifying fibromas and fibrous cortical defects. Therefore, in rare cases like this one, clinical and radiological findings take precedence over histological confirmation, unlike most other bone tumors [11].

Treatment for BFH typically involves complete tumor excision through curettage, followed by bone grafting. Extended curettage with phenol application is another option to enhance tumor removal. Bone defects can be stabilized using autologous bone grafts, allogeneic bone grafts, or polymethyl methacrylate bone cement [4]. Additional stabilization using plates may be necessary depending on the tumor’s location [3].

Each treatment method has its own benefits and drawbacks. Autologous bone grafts provide permanent reconstruction once fully integrated but are limited in quantity and may cause donor site morbidity. Allogeneic bone grafts are costly and difficult to obtain in resource-limited settings. Bone cement offers immediate structural support and simplifies radiographic monitoring for recurrence, but its non-biological nature makes it prone to fractures. Additionally, cementing increases the risk of arthrosis due to potential articular cartilage degeneration, especially in subchondral lesions located in weight-bearing areas [12].

In our case, we performed extended curettage with phenol. Since the lesion encompassed almost the entire calcaneus, a weight-bearing bone, we opted for a combination of autologous fibular strut grafts, iliac crest bone grafts, and artificial bone grafts in the form of synthetic hydroxyapatite granules to fill the defect. This approach was chosen to achieve optimal stability and enhanced mechanical strength. Allogeneic bone grafts were not feasible due to their high cost and the lack of availability from bone banks.

Harvesting autologous non-vascularized fibular strut grafts is a straightforward, quick procedure that has been successfully used for reconstructing bone defects after tumor resection in areas such as the humerus, radius, pelvis, femur, and tibia. It offers a biological reconstruction option with good long-term outcomes and low rates of donor-site complications [13]. Studies have demonstrated that fibular strut grafts have a significantly lower rate of non-oncological complications and a comparable rate of tumor recurrence when compared to bone cement [14]. Additionally, the robust nature of this construct eliminated the need for supplementary hardware such as plates and screws.

BFH generally has a favorable prognosis when treated with appropriate curettage and bone grafting, although cases of recurrence and pulmonary metastasis have been documented [15]. Fortunately, no such complications were observed in our patient during the five-year follow-up visit.

## Conclusions

BFH of the calcaneus is an extremely rare tumor, making accurate diagnosis crucial. Clinico-radiological correlation is more significant than histological findings in establishing the diagnosis. The preferred treatment approach involves adequate curettage followed by bone grafting. Although fibular strut grafts are rarely used for calcaneal bone defects, this case is the first to report the use of autologous non-vascularized free fibular strut grafts for managing a calcaneal bone defect following BFH curettage.

Given that the lesion involved nearly the entire calcaneus, the use of autologous fibular strut grafts provided enhanced stability and created a sturdy, mechanically robust construct, enabling early weight-bearing for the patient. Additionally, this approach offers a cost-effective alternative to allogeneic bone grafts and reduces the need for further stabilization with implants like plates and screws.
